# Anti-inflammatory effect of simvastatin in an experimental model of spinal cord trauma: involvement of PPAR-α

**DOI:** 10.1186/1742-2094-9-81

**Published:** 2012-04-26

**Authors:** Emanuela Esposito, Barbara Rinaldi, Emanuela Mazzon, Maria Donniacuo, Daniela Impellizzeri, Irene Paterniti, Annalisa Capuano, Placido Bramanti, Salvatore Cuzzocrea

**Affiliations:** 1 Department of Clinical and Experimental Medicine and Pharmacology, School of Medicine, University of Messina, 98125 Messina, Italy; 2 Department of Experimental Medicine, Second University of Naples, via Costantinopoli 16, 80138 Naples, Italy; 3 IRCCS Centro Neurolesi "Bonino-Pulejo", via Provinciale Palermo, C. da Casazza, 98124, Messina, Italy

**Keywords:** SCI, PPAR-α, simvastatin, inflammation

## Abstract

**Background:**

Statins such as simvastatin are inhibitors of 3-hydroxy-3-methylglutaryl-coenzyme A (HMG-CoA) reductase used in the prevention of cardiovascular disease. In addition to their cholesterol-lowering activities, statins exert pleiotropic anti-inflammatory effects, which might contribute to their beneficial effects on lipid-unrelated inflammatory diseases. Recently it has been demonstrated that the peroxisome proliferator-activated receptor (PPAR)-α mediates anti-inflammatory effects of simvastatin in vivo models of acute inflammation. Moreover, previous results suggest that PPAR-α plays a role in control of secondary inflammatory process associated with spinal cord injury (SCI).

**Methods:**

With the aim to characterize the role of PPAR-α in simvastatin activity, we tested the efficacy of simvastatin (10 mg/kg dissolved in saline i.p. 1 h and 6 h after the trauma) in an experimental model of SCI induced in mice by extradural compression of the spinal cord (T6-T7 level) using an aneurysm clip with a closing force of 24 g via a four-level T5-T8 laminectomy, and comparing mice lacking PPAR-α (PPAR-α KO) with wild type (WT) mice. In order to elucidate whether the effects of simvastatin are due to activation of the PPAR-α, we also investigated the effect of a PPAR-α antagonist, GW6471 (1 mg/kg administered i.p. 30 min prior treatment with simvastatin) on the protective effects of on simvastatin.

**Results:**

Results indicate that simvastatin activity is weakened in PPAR-α KO mice, as compared to WT controls. In particular, simvastatin was less effective in PPAR-α KO, compared to WT mice, as evaluated by inhibition of the degree of spinal cord inflammation, neutrophil infiltration, nitrotyrosine formation, pro-inflammmatory cytokine expression, nuclear factor (NF)-κB activation, inducible nitric-oxide synthase (iNOS) expression, and apoptosis. In addition we demonstrated that GW6471 significantly antagonized the effect of the statin and thus abolished the protective effect.

**Conclusions:**

This study indicates that PPAR-α can contribute to the anti-inflammatory activity of simvastatin in SCI.

## Introduction

Spinal cord injury (SCI) is the result of an initial physical trauma followed by a secondary degenerative process. SCI leads to the destruction of ascending and descending axonal tracts that control motor, sensory and autonomic functions. The level of SCI is an important factor because the consequences are devastating. Patients suffer from permanent, often lifelong motor and sensory disabilities below the site of injury, combined with impaired basic vital functions. Functional recovery is restricted because axons in the central nervous system (CNS) regenerate poorly. Post-traumatic inflammatory reaction may play an important role in the secondary injury processes after SCI [1,2].

In particular the secondary damage is determined by a large number of cellular, molecular, and biochemical cascades and a large body of data suggests the presence of a local inflammatory response, which amplifies the secondary damage [3].

The contemporary management of SCI consists of supportive care and stabilization of the spine [4]. Numerous pharmacologic therapies have been evaluated for the treatment of SCI, although none have met with substantial success.

Statins, such as simvastatin, are lipid-lowering drugs that inhibit 3-hydroxy-3-methylglutaryl co-enzyme A (HMG-CoA) reductase. They are widely prescribed cholesterol-lowering drugs and are the first-line treatment for the prevention of coronary artery disease and atherosclerosis [5]. Simvastatin have been shown to exhibit important immunomodulatory and anti-inflammatory effects independent of lipid lowering [6,7]. Statins have pleiotropic effects on endothelium, platelets, smooth muscle cells and inflammation. These effects included the improvement of endothelial and microvascular function, the reduction of inflammation through decreased expression of pro-inflammatory transcriptional factors such as Nuclear Factor (NF)-κB that in turn decreases cytokines, chemokines and nitric oxide synthase (iNOS) expression [8]. In particular, previous studies have shown that simvastatin inhibits nitric oxide (NO) production through a reduction of iNOS in a model of endotoxic shock in rats [9].

Moreover, a large and growing body of evidences also suggest beneficial therapeutic activities of statins in immune and inflammatory diseases such as multiple sclerosis, Alzheimer's disease, ischemic stroke, transplant rejection, rheumatoid arthritis, and asthma [10,11]. Several clinical observations indicate that these effects cannot be attributed to their cholesterol-lowering activities [12].

Peroxisome proliferator-activated receptors (PPARs) are members of the nuclear hormone receptor superfamily of ligand-activated transcription factors [13]. PPARs regulate target gene expression by binding as heterodimers with retinoid × receptors (RXRs) to specific peroxisome proliferator response elements (PPREs) in enhancer sites of regulated genes of DNA. The PPAR subfamily comprises three members, PPAR-α PPAR-β and PPAR-γ [14]. RXRs are also members of the nuclear hormone receptor superfamily that are activated by binding of 9-cis retinoic acid [15]. In the absence of a ligand, high affinity complexes are formed between the PPAR-RXR heterodimer and nuclear receptor co-repressor proteins, preventing transcriptional activation by sequestration of the nuclear receptor heterodimer from the promoter. Binding of a ligand to the heterodimer results in the release of the co-repressor from the complex, which in turn results in the binding of the activated heterodimer to the response element in the promoter region of the relevant target genes, resulting in either the activation or suppression of a specific gene [15,16].

In rats, PPAR-α is most highly expressed in brown adipose tissue, followed by liver, kidney, heart and skeletal muscle [17]. The presence of PPAR-α in discrete areas of brain and spinal cord has been suggested [18], although its role remains unknown.

PPAR-α binds to a diverse set of ligands, namely, arachidonic acid metabolites (prostaglandins and leukotrienes) and plasticizers and synthetic fibrate drugs including clofibrate, fenofibrate and bezafibrate [19]. Given that no single high affinity natural ligand has been identified for PPAR-α, it has been proposed that a physiological role of the receptor may be to sense the total flux of dietary fatty acids in key tissues. Many PPAR-α ligands, including most of the common fibrate ligands, show only modest selectivity over the other PPAR subtypes. However, a potent thioisobutyric acid GW7647 has been identified that shows excellent selectivity for both murine and human PPAR-α [20].

Various lines of evidence suggest that the activation of PPAR-α by synthetic agonists causes marked anti-inflammatory effects in experimental models [21-23]. Indeed, we have recently demonstrated using PPAR-α knock-out (PPAR-αKO) mice that endogenous PPAR-α activity reduces the degree of development of inflammation and tissue injury events associated with spinal cord trauma in mice, suggesting the existence of an intrinsic anti-inflammatory mechanism mediated by PPAR-α [24]. In addition, it has been reported that PPAR-α activation can result in inhibition of NF-κB activation and inflammatory genes expression [25-27].

Moreover, recently, Paumelle and colleagues have clearly demonstrated that the acute anti-inflammatory effect of simvastatin occurs via PPAR-α by a mechanism involving inhibition of PKC-α inactivation of PPAR-α transrepression activity [28].

To characterize the role of PPAR-α in statins-mediated anti-inflammatory activity, we tested the efficacy of simvastatin in an experimental model of spinal cord trauma induced in mice by the application of vascular clips (force of 24 g) to the dura via a four-level T5-T8 laminectomy, comparing PPAR-αKO and WT mice.

## Methods

### Animals

Mice (6 weeks old, 20-22 g) with a targeted disruption of the PPAR-α gene (PPAR-αKO) and littermate wild-type controls (PPAR-αWT) were purchased from Jackson Laboratories (Harlan Nossan, Italy). Mice homozygous for the Pparat^niJ^Gonz targeted mutation mice are viable, fertile and appear normal in appearance and behaviour. Exon eight, encoding the ligand-binding domain, was disrupted by the insertion of a 1.14 kb neomycin resistance gene in the opposite transcriptional direction. After electroporation of the targeting construct into J1 ES cells, the ES cells were injected into C57BL/6N blastocysts. This stain was created on B6,129S4 background and is maintained as a homozygote on a 129S4/SvJae background by brother sister mating. The animals were housed in a controlled environment and provided with standard rodent chow and water. The study was approved by the University of Messina Animal Care Review Board. Animal care was in compliance with Italian regulations on protection of animals used for experimental and other scientific purposes (D.M. 116192) as well as with the EEC regulations (O.J. of E.C. L 358/1 12/18/1986).

### SCI

Mice were anaesthetized using chloral hydrate (40 μg/kg body weight). A longitudinal incision was made on the midline of the back, exposing the paravertebral muscles. These muscles were dissected away exposing T5-T8 vertebrae. The spinal cord was exposed via a four-level T5-T8 laminectomy and SCI was produced by extradural compression of the spinal cord (T6-T7) using an aneurysm clip with a closing force of 24 g as previously described [24]. Following surgery, 1 ml of saline was administered subcutaneously in order to replace the blood volume lost during surgery. During the surgery and the recovery from anaesthesia, the mice were placed on a warm heating pad and covered with a warm towel. The mice were singly housed in a temperature-controlled room at 27°C for a survival period of 10 days. Food and water were provided to the mice *ad libitum*. During this time period, the animals' bladders were manually voided twice a day until the mice were able to regain normal bladder function. In all injured groups, the spinal cord was compressed for 1 min. Sham injured animals were only subjected to laminectomy.

### Experimental groups

#### Mice were randomly allocated into the following groups

(i) *PPAR-αWT SCI group*. WT mice were subjected to SCI (*N *= 30); (ii) *PPAR-αKO SCI group*. PPAR-αKO mice were subjected to SCI (*N *= 30); (iii) *PPAR-αWT SCI+*1 h and 6 h after SCI *Simvastatin group*. Identical to the *PPAR-αWT SCI group *but simvastatin (10 mg/kg dissolved in saline i.p. bolus) was administered as an intraperitoneal (i.p.) injection at 1 h and 6 h after SCI (*N *= 30); (iv) *PPAR-αKO simvastatin group*. Identical to the *PPAR-αKO SCI group *but simvastatin (10 mg/kg dissolved in saline i.p. bolus) which was administered as an i.p. injection starting 1 h and 6 h after SCI (*N *= 30); (v) *PPAR-αWT Sham + saline group*. Mice were subjected to the surgical procedures as above group except that the aneurysm clip was not applied (*N *= 30); (vi) *PPAR-αKO Sham + saline group*. Mice were subjected to the surgical procedures as above group except that the aneurysm clip was not applied (*N *= 30); (vii) *PPAR-αWT Sham + simvastatin group*. Identical to *PPAR-αWT Sham+saline group *except for the administration of simvastatin (*N *= 30); (viii) *PPAR-αKO Sham + simvastatin group*. Identical to *PPAR-αKO Sham+saline group *except for the administration of simvastatin (*N *= 30); (ix) *PPAR-αWT SCI + GW6471 group*. Identical to the *PPAR-αWT SCI group *but GW6471 was administered (1 mg/kg i.p. bolus) 30 min, and 5 hours and 30 min after SCI (*N *= 30); (x) *PPAR-αWT SCI + GW6471 *+ *Simvastatin group*. Identical to the *PPAR-αWT Simvastatin group*, but GW6471 was administered (1 mg/kg i.p. bolus) 30 min prior to simvastatin. The doses of simvastatin (10 mg/kg) and GW6471 (1 mg/kg) were chosen in agreement with previous studies [29,30].

### Light microscopy

Spinal cord tissues from perilesional zone were taken at 24 h following trauma. Tissue segments containing the lesion (1 cm on each side of the lesion) were paraffin embedded and cut into 5-μm-thick sections. The tissue segments were fixed for 24 h in paraformaldehyde solution (4% in PBS 0.1 M) at room temperature, dehydrated by graded ethanol and embedded in Paraplast (Sherwood Medical, Mahwah, NJ). Tissue sections were deparaffinized with xylene, stained with Haematoxylin/Eosin (H&E) and Luxol Fast Blue staining (used to assess demyelination) and studied using light microscopy connected to an Imaging system (AxioVision, Zeiss, Milan, Italy).

The histopathological changes of the gray matter were scored on a 6-point scale: 0, no lesion observed, 1, gray matter contained 1 to 5 eosinophilic neurons; 2, gray matter contained 5 to 10 eosinophilic neurons; 3, gray matter contained more than 10 eosinophilic neurons; 4, small infarction (less than one third of the gray matter area); 5, moderate infarction; (one third to one half of the gray matter area); 6, large infarction (more than half of the gray matter area). The scores from all the sections from each spinal cord were averaged to give a final score for individual mice. All the histological studies were performed in a blinded fashion.

### Terminal Deoxynucleotidyltransferase-Mediated UTP End Labelling (TUNEL) Assay

TUNEL assay was conducted by using a TUNEL detection kit according to the manufacturer's instruction (Apotag, HRP kit DBA, Milan, Italy). Briefly, sections were incubated with 15 μg/ml proteinase K for 15 min at room temperature and then washed with PBS. Endogenous peroxidase was inactivated by 3% H_2_O_2 _for 5 min at room temperature and then washed with PBS. Sections were immersed in terminal deoxynucleotidyltransferase (TdT) buffer containing deoxynucleotidyl transferase and biotinylated dUTP in TdT buffer, incubated in a humid atmosphere at 37°C for 90 min, and then washed with PBS. The sections were incubated at room temperature for 30 min with anti-horseradish peroxidase-conjugated antibody, and the signals were visualized with diaminobenzidine. The number of TUNEL positive cells/high-power field was counted in 5 to 10 fields for each coded slide.

### Myeloperoxidase activity

Myeloperoxidase (MPO) activity, an indicator of polymorphonuclear leukocyte (PMN) accumulation, was determined as previously described [31]. At the specified time following SCI, spinal cord tissues were obtained and weighed, each piece homogenized in a solution containing 0.5% (w/v) hexadecyltrimethyl-ammonium bromide dissolved in 10 mM potassium phosphate buffer (pH 7) and centrifuged for 30 min at 20,000 × g at 4°C. An aliquot of the supernatant was then allowed to react with a solution of tetramethylbenzidine (1.6 mM) and 0.1 mM hydrogen peroxide. The rate of change in absorbance was measured spectrophotometrically at 650 nm. MPO activity was defined as the quantity of enzyme degrading 1 μmol of peroxide/min at 37°C and was expressed in units per g of wet tissue.

### Grading of motor disturbance

The motor function of mice subjected to compression trauma was assessed once a day for 10 days after injury. Recovery from motor disturbance was graded using the Basso Mouse Scale (BMS) [32].

### Measurement of Tumor Necrosis Factor-α and Interleuchin (IL)-1β concentration

Portions of spinal cord tissues from perilesional zone, collected 24 h after SCI, were homogenized in PBS containing 2 mmol/L of PMSF (Sigma Chemical Co.), and tissue TNF-α and IL-1β levels were evaluated. The assay was carried out by using a colorimetric, commercial kit (Quantikine R&D System, USA) according to the manufacturer instructions. All TNF-α and IL-1β determinations were performed in duplicate serial dilutions.

### Localization of nitrotyrosine, TNF-α, Bax, iNOS, Bcl-2, Fas-ligand, nitrotyrosine, by immunohistochemistry

At the 24^th ^hour after SCI, the tissues were fixed in 10% (w/v) PBS-buffered formaldehyde and 5 μm sections were prepared from paraffin embedded tissues. After deparaffinization, endogenous peroxidase was quenched with 0.3% H_2_O_2 _in 60% methanol for 30 min. The sections were permeabilized with 0.1% Triton X-100 in PBS for 20 min. Non-specific adsorption was minimized by incubating the section in 2% normal goat serum in PBS for 20 min. Endogenous biotin or avidin binding sites were blocked by sequential incubation for 15 min with avidin and biotin (DBA, Milan, Italy). Sections were incubated overnight with anti-nitrotyrosine antibody (1:500 in PBS, Upstate), with anti-Bax antibody (1:500 in PBS, Santa Cruz Biotechnology) with anti-TNF-α polyclonal antibody (1:100 in PBS, Santa Cruz Biotechnology) with anti-iNOS (1:500 in PBS, Transduction Laboratories), or with anti-Bcl-2 antibody (1:100 in PBS, Santa Cruz Biotechnology), with anti-Fas-ligand antibody (1:100 in PBS, Abcam). Specific labelling was detected with a biotin-conjugated goat anti-rabbit, donkey anti-goat or goat anti-mouse IgG and avidin-biotin peroxidase complex (DBA, Milan, Italy). To verify the binding specificity for Bax, Bcl-2, iNOS, Fas-ligand, TNF-α or nitrotyrosine, some sections were also incubated with primary antibody only (no secondary antibody) or with secondary antibody only (no primary antibody). In these situations, no positive staining was found in the sections indicating that the immunoreactions were positive in all the experiments carried out.

Immunocytochemistry photographs (*N *= 5) were assessed by densitometry as previously described [33] by using Imaging Densitometer (AxioVision, Zeiss, Milan, Italy) and a computer program. In particular the densitometry analysis was carried out in section in which the spinal cord tissues were orientated longitudinally in order to observe all the histological portions.

### Nuclear and cytoplasm protein extraction

Spinal cord Tissues from perilesional zone were obtained from the animals of all experimental groups were homogenized on ice in ice-cold hypotonic *buffer A *(10 mM Hepes pH 7.9, 10 mM KCl, 0.1 mM EDTA, 0.1 mM EGTA, 1 mM DTT, 0.5 mM PMSF with a protease inhibitor cocktail) using a Politron PT 13,000 D tissue homogenizer (Kinematica). After 15 min incubation on ice, the homogenates were centrifuged at 1,000 g for 10 min at 4°C. Supernatants containing cytoplasm extracts were stored at -80°C. Nuclear pellets were resuspended in ice-cold *buffer B *(1% Triton X-100, 150 mM NaCl, 10 mM TRIS-HCl pH 7.4, 1 mM EGTA, 1 mM EDTA, 0,2 mM PMSF, 20 mm leupeptin, 0,2 mM sodium orthovanadate) and the tubes were vigorously rocked at 4°C for 30 min on a shaking platform. The nuclear extracts were centrifuged at 13,000 g for 15 min at 4°C. The supernatants were frozen in aliquots at -80°C until use. Protein concentrations were determined by the Bradford method using bovine serum albumin (BSA) as standard [34].

### NF-κB nuclear translocation assessment and PPAR-α expression by Western blot

Proteins from cytoplasm and nuclear fraction were added to sample buffer [0.125 M Tris-HCl, (pH 6.8), 4% SDS, 20% glycerol, 10% β-mercaptoethanol, 0.004% bromphenol blue], and boiled in a water bath for 5 min. Protein samples (40 μg per lane) were separated on denaturing 12% SDS polyacrylamide gel and transferred to a nitrocellulose membrane. Non-specific binding to the membrane was blocked for 1 h at room temperature with 5% milk in PBS. Membranes were then incubated at 4°C overnight with primary antibody in milk-PBS- Tween 20 0.1% (PMT) for NF-κB p65 (1:1000; Santa Cruz Biotechnology), PPAR-α, (Santa Cruz Biotechnology, 1:500), and IκB-α (1:1000; Santa Cruz Biotechnology) in PMT, washed three times with PBS -0.1% Tween, and then incubated for 1 h at room temperature with a secondary antibody (peroxidase-conjugated bovine anti-mouse IgG secondary antibody or peroxidase-conjugated goat anti-rabbit IgG, 1:2000; Jackson ImmunoResearch, West Grove, PA). Polyclonal anti-actin and anti-lamin A/C antibodies were used as an internal standard for cytoplasm and nuclear fractions, respectively. The immunoreactive bands were visualized using an enhanced chemilumunescence system (SuperSignal West Femto Maximum Sensitivity Substrate, Pierce). The protein bands were scanned and quantitated with Gel Doc-2000 (Bio-Rad).

### Reagents

Biotin blocking kit, biotin-conjugated goat anti-rabbit IgG and avidin-biotin peroxidase complex were obtained from Vector Laboratories (Burlingame, CA, USA). Reagents and secondary and nonspecific IgG antibody for immunohistochemical analysis were from Vector Laboratories InC. PPAR-alpha antagonist N-((2S)-2-(((1Z)-1-Methyl-3-oxo-3-(4-(trifluoromethyl)phenyl)prop-1-enyl)amino)-3-(4-(2-(5-methyl-2-phenyl-1,3-oxazol-4-yl)ethoxy)phenyl)propyl)propanamide (GW6471) was purchased by Tocris. All other reagents and compounds used were obtained from Sigma Chemical Company.

### Statistical analysis

All values in the figures and text are expressed as mean ± standard error of the mean (SEM) of N observations. For the in vivo studies N represents the number of animals studied. In the experiments involving histology or immunohistochemistry, the figures shown are representative of at least three experiments performed on different experimental days. The results were analyzed by one-way ANOVA followed by a Bonferroni post-hoc test for multiple comparisons. A p-value of less than 0.05 was considered significant. BMS scale data were analyzed by the Mann-Whitney test and considered significant when p- value was < 0.05.

## Results

### Effects of simvastatin treatment on PPAR-α expression in spinal cord tissue

**Figure 1:**
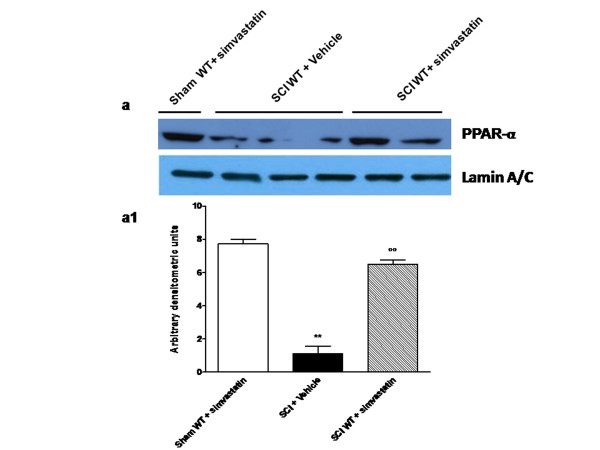
**PPAR-α expression in spinal cord tissues**. A basal level of PPAR-α was detected in the spinal cord tissues from shamWT-operated mice, whereas in SCI-operated PPARαWT mice the levels were substantially reduced (**panels A and A1**). Simvastatin treatment significantly reversed the spinal PPAR-α levels down-regulated by SCI (**panels A and A1**). The relative expression of the protein bands was standardized for densitometric analysis to lamin levels, reported in panel **a1**, and expressed as mean ± s.e.m. from n = 5/6 spinal cord for each group. *P < 0.01 *versus *sham, °*P *< 0.01 *versus *SCI+vehicle.

Previous studies have demonstrated an important role for PPAR-α in SCI [35] and have suggested that the ability of simvastatin to reduce inflammation is also dependent on activation of PPAR-α [36,37]. Thus we evaluated PPAR-α expression in the nuclear fractions from spinal cord tissue by Western Blot analysis. A basal level of PPAR-α was detected in spinal cord tissue from sham WT-simvastatin treated mice, whereas SCI significantly reduced the levels of PPAR-α in the spinal cords (**p < 0.01; Figure 1 panels a and a1). Administration of simvastatin, 1 h and 6 h after SCI, significantly reversed spinal cord PPAR-α levels down-regulated by SCI (##*p *< 0.01), as determined by densitometric analysis (Figure 1).

### Role of functional PPAR-α gene in the anti-inflammatory effect of simvastatin on the degree of spinal cord trauma

**Figure 2:**
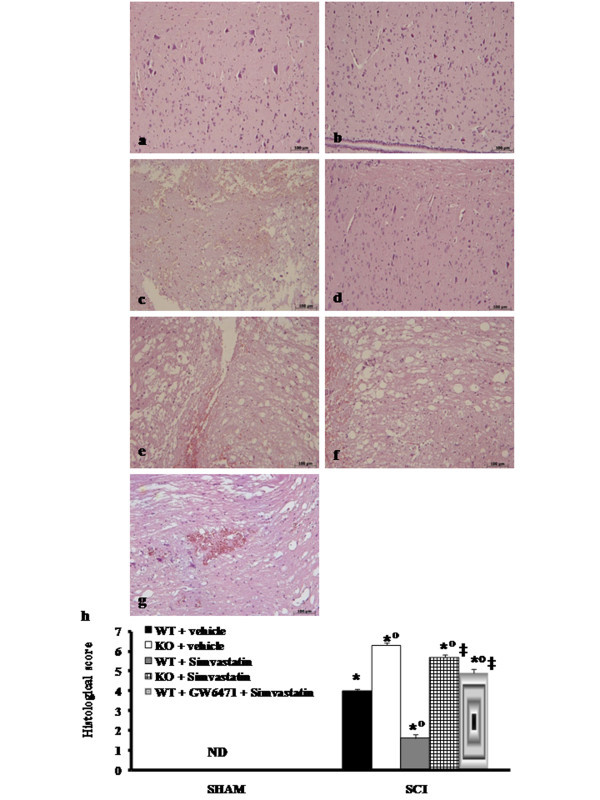
**Effect of PPAR-α on the anti-inflammatory property of simvastatin on the degree of SCI**. No histological alterations have been found in the spinal cord tissue collected from PPAR-α WT or PPAR-α KO sham-operated mice (a,b and histological score h). A significant damage to the spinal cord from PPAR-αWT mice at the perilesional zone was observed from H&E staining (c see histological score h). The treatment of PPAR-αWT with simvastatin resulted in a significant decrease in the extent and severity of the histological signs (d, and histological score h). The absence of PPAR-α gene significantly increases the extent and severity of the histological signs (e, and histological score h). The genetic absence of the PPAR-α receptor (f) as well as the treatment with GW6471 (g) significantly blocked the effect of the simvastatin treatment (histological score h**)**. Data are means ± SEM of 10 mice for each group. *P < 0.01 *vs*. Sham; °*P *< 0.01 *vs*. SCI-WT group; ‡*P *< 0.01 *vs*. simvastatin -treated -WT group. ND: not detectable.

Twenty four hours after the trauma a significant damage to the spinal cord from WT mice at the perilesional zone was observed in H&E staining slices as assessed by the presence of oedema as well as an alteration of the white matter (Figure 2c and see histological score h) when compared with spinal cord tissue collected from PPAR-α WT (Figure 2a). No histological alteration was observed in spinal cord tissue from vehicle-treated PPAR-αKO mice (Figure 2b; see histological score h). Simvastatin treatment resulted in a significant decrease in the extent and severity of the histological signs of spinal cord trauma (Figure 2d and see histological score h). The absence of PPAR-α gene significantly increases the extent and severity of trauma in the spinal cord tissue (Figure 2e see histological score h). The genetic absence of the PPAR-α significantly blocked the effect of the simvastatin treatment (Figure 2f see histological score h**)**. Moreover, treatment with the PPAR-α antagonist GW6471 (1 mg/kg) blocked simvastatin-mediated neuroprotection (Figure 2g, see histological score h).

**Figure 3:**
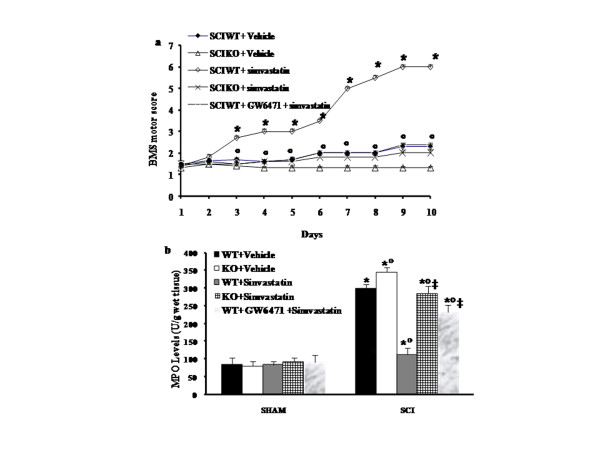
**Role of PPAR-α in simvastatin-induced recovery of motor function and inhibition of neutrophil infiltration after SCI**. To evaluate if histological damage to the spinal cord was associated a loss of motor function the modified BMS hind limb locomotor rating scale score was evaluated (a). The degree of motor disturbance was assessed every day until 10 days after SCI by BMS criteria. Treatment of injured- PPAR-α WT mice with simvastatin significantly reduces the motor disturbance after SCI (a). In addition, we investigate the role of simvastatin on the neutrophil infiltration by measurement of the activity of MPO. Treatment with simvastatin significantly reduced the MPO activity (h). The genetic absence of the PPAR-α as well as the treatment with GW6471 significantly blocked the effect of simvastatin treatment on motor recovery (a) and neutrophils infiltration (b). Data are means ± SEM of 10 mice for each group. *P < 0.01 *vs*. Sham; °*P *< 0.01 *vs*. SCI-WT group; ‡*P *< 0.01 *vs*. simvastatin -treated WT mice.

In order to evaluate if histological damage to the spinal cord was associated a loss of motor function the BMS hind limb locomotor rating scale score was evaluated (Figure 3a). While motor function was not impaired in sham mice (data not shown), PPAR-α WT mice undergoing SCI had significant deficits in hind limb movement (Figure 3a). In contrast, a significant worsened of hind limb motor disturbances was observed in PPAR-αKO operated mice (Figure 3a). The treatment of PPAR-α WT with simvastatin resulted in a significant recovery of motor function (Figure 3a). The genetic absence of the PPAR-α significantly blocked also the effect of the simvastatin treatment on improvement of motor function (Figure 3a**)**.

In addition, in order to evaluate that the protective effects of simvastatin on the severity of spinal cord trauma are due to agonistic actions on the PPAR-α receptor, we have also investigated the effect of GW6471, a PPAR-α antagonist. Co-administration of GW6471 and simvastatin significantly blocked the effect of the statin on tissue injury (Figure 1g see histological score h) and motor function impairment (Figure 3a) induced by SCI.

### Role of PPAR-α in simvastatin-induced inhibition of PMN infiltration

The above histological pattern of spinal cord injury appeared to be correlated with the influx of leukocytes into the spinal cord. Therefore, we investigate the role of PPAR-α ligand on the neutrophils infiltration by measurement of the activity MPO. MPO activity was significantly elevated (p < 0.001) at 24^th ^hour after SCI in PPAR-αWT mice (Figure 3b). In PPAR-αKO mice following SCI spinal cord MPO activity was significantly enhanced (p < 0.01) in comparison to those of WT animals (Figure 3b). On the contrary, simvastatin significantly reduced the degree of PMN infiltration (determined as increase in MPO activity) in spinal cord tissues collected at 24^th ^hour after SCI in WT mice **(**Figure 3b). The genetic absence of the PPAR-α receptor as well as the treatment with GW6471, a PPAR-α antagonist significantly blocked the effect of the simvastatin on the neutrophils infiltration (Figure 3b).

### Role of PPAR-α in simvastatin-induced inhibition of tnf-α and il-1β after spinal cord trauma

**Figure 4:**
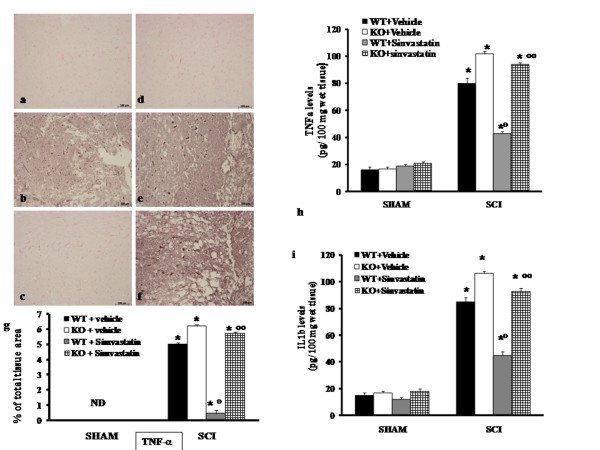
**Role of PPAR-α in simvastatin-induced inhibition of TNF-α and IL-1β after SCI WT mice show a significant production of cytokines at 24 hours after SCI (h, i)**. Cytokine levels were significantly enhanced in injured-PPAR-α KO mice (h, i). Treatment with simvastatin significantly reduced the spinal cord TNF-α and IL-1β (i) production. The genetic absence of the PPAR-α receptor significantly blocked the effect of simvastatin treatment (h, i). Data are means **±**S.E. mean of 10 mice for each group. *P < 0.01 *vs*. Sham; °*P *< 0.01 *vs*. SCI-WT group; °°*P *< 0.01 *vs*. simvastatin-treated WT group. In addition, tissue sections obtained from PPAR-αWT animals at 24 h after SCI demonstrate positive staining for TNF-α when compared with spinal cord tissue collected from PPAR-α sham-operated mice (a, d and g) The intensity of the positive staining for TNF-α was markedly increased in tissue section from injured-PPAR-αKO mice (e, g). Section from simvastatin-treated PPAR-αWT mice did not reveal positive staining for TNF-α (c, g) The genetic absence of the PPAR-α significantly blocked the effect of simvastatin on the TNF-α expression (f, g). Data are means ± SEM of 10 mice for each group. The assay was carried out by using Imaging Densitometer (AxioVision, Zeiss, Milan, Italy). Data are expressed as % of total tissue area. This figure is representative of at least 3 experiments performed on different experimental days. *P < 0.01 vs. Sham; °P < 0.01 vs. SCI-WT group; °°P < 0.01 vs. simvastatin-treated WT mice. ND: not detectable.

Release of pro-inflammatory cytokines is an important mechanism responsible for spinal cord trauma. A substantial increase in TNF-α and IL-1β formation was found in spinal cord tissues collected from WT mice at 24 h after SCI (Figure 4h, i). Spinal cord levels of TNF-α and IL-1β were significantly higher in PPAR-α deficient mice in comparison to those of WT animals **(**Figure 4h, i). In contrast a significant inhibition of TNF-α and IL-1β levels was observed in the spinal cord tissues collected from of WT mice treated with simvastatin (Figure 4h, i). The genetic absence of the PPAR-α receptor significantly blocked the effect of the simvastatin on the production of pro-inflammatory cytokines (Figure 4h, i). In addition, tissue sections obtained from WT animals at 24 h after SCI demonstrate positive staining for TNF-α (Figure 4b see densitometry analysis g) mainly localized in inflammatory cells as well as in nuclei of Schwann cells in the white and gray matter of the spinal cord tissues. In SCI-injured PPAR-αKO mice, the staining for TNF-α was visibly and significantly increased in comparison with the WT mice (Figure 4e and see densitometry analysis g). Section from simvastatin-treated PPAR-αWT mice did not reveal positive staining TNF-α (Figure 4c and see densitometry analysis g**)**. The genetic absence of the PPAR-α receptor significantly blocked the effect of simvastatin on the TNF-α expression (Figure 4f and see densitometry analysis g). Sections of spinal cord tissues from sham-administered PPAR-α mice did not stain for TNF-α (Figure 4a, d and see densitometry analysis g).

### Role of PPAR-α in simvastatin-induced inhibition of iNOS expression

**Figure 5:**
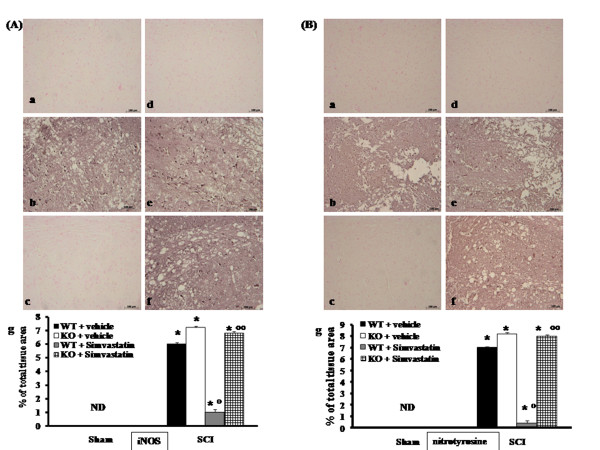
**Role of PPAR-α in simvastatin-induced inhibition on iNOS expression and on nitrotyrosine formation**. Spinal cord from sham PPAR-αWT mice did not stain for iNOS (panel A a, d and g). Spinal cord sections from injured-PPAR-α WT and from injured-PPAR-α mice (panel A e, g) exhibited positive staining for iNOS (panel A b, g); section from simvastatin-treated PPAR-αWT SCI mice did not reveal any positive staining for iNOS (panel A c, g). The genetic absence of the PPAR-α significantly blocked the effect of the simvastatin on iNOS expression (panel A f, g). In addition, immunohistochemical analysis for nitrotyrosine show positive staining localized in the inflammatory cells in the injured area from SCI-PPAR-αWT mice (panel B b, g). The intensity of the positive staining for nitrotyrosine was markedly increased in tissue section obtained from injured-PPAR-αKO mice (panel B e, g). No positive staining for nitrotyrosine was observed in tissue section from simvastatin-treated PPAR-αWT mice (panel B c, g). The genetic absence of the PPAR-α significantly blocked the effect of simvastatin treatment (panel B f, g). Densitometry analysis of immunocytochemistry photographs (n = 5 photos from each sample collected from all mice in each experimental group) for iNOS (panel A, g) and nitrotyrosine (panel B, g) was assessed. The assay was carried out by using Imaging Densitometer (AxioVision, Zeiss, Milan, Italy). Data are expressed as % of total tissue area. This figure is representative of at least 3 experiments performed on different experimental days. *P < 0.01 *vs*. Sham; °*P *< 0.01 *vs*. SCI-WT group; °°*P *< 0.01 *vs*. simvastatin-treated WT group. ND: not detectable.

Spinal cord sections from sham PPAR-α mice did not stain for iNOS (Figure 5Aa, Ad and see densitometry analysis Ag). Spinal cord sections obtained from vehicle-treated SCI-PPAR-α WT mice exhibited positive staining for iNOS (Figure 5Ab and see densitometry analysis Ag) mainly localized in inflammatory cells as well as in nuclei of Schwann cells in the white and gray matter of the spinal cord tissues. In SCI-injured PPAR-αKO mice, the staining for iNOS was visibly and significantly increased (Figure 5Ae and see densitometry analysis Ag) in comparison with the PPAR-αWT mice. Section from simvastatin-treated WT mice did not reveal any positive staining for iNOS (Figure 5Ac and see densitometry analysis Ag). The genetic absence of the PPAR-α receptor significantly blocked the effect of the simvastatin on iNOS expression (Figure 5Af and see densitometry analysis Ag).

### Role of PPAR-α in simvastatin-induced inhibition of nitrotyrosine formation

To determine the localization of *"peroxynitrite formation" *and/or other nitrogen derivatives produced after SCI, nitrotyrosine, a specific marker of nitrosative stress, was measured by immunohistochemical analysis in the spinal cord tissues. Sections of spinal cord from sham PPAR-α mice did not stain for nitrotyrosine (Figure 5Ba, Bd and see densitometry analysis Bg). Spinal cord sections obtained from vehicle-treated SCI-WT mice exhibited positive staining for nitrotyrosine (Figure 5Bb, and see densitometry analysis Bg) mainly localized in inflammatory cells as well as in nuclei of Schwann cells in the white and gray matter of the spinal cord tissues. In SCI-injured PPAR-αKO mice, the staining for nitrotyrosine was visibly increased (Figure 5Be and see densitometry analysis Bg) in comparison with the PPAR-αWT mice. Section from simvastatin-treated WT mice did not reveal any positive staining for nitrotyrosine (Figure 5Bc, and see densitometry analysis Bg). The genetic absence of the PPAR-α receptor significantly blocked the effect of the simvastatin on nitrotyrosine formation (Figure 5Bf and see densitometry analysis Bg).

### Role of PPAR-α in simvastatin-induced inhibition of Fas-ligand expression

**Figure 6:**
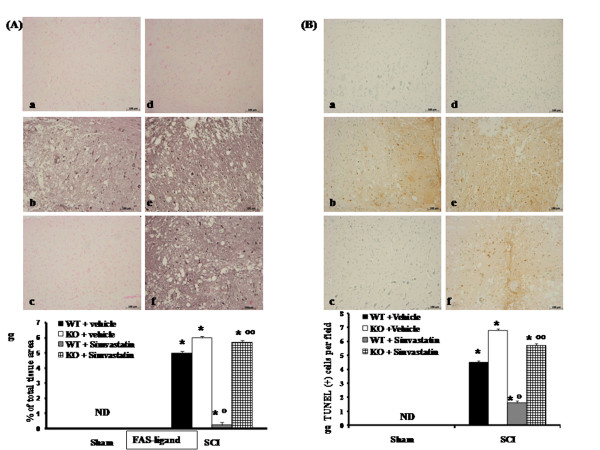
**Immunohistochemical localization of Fas-ligand and TUNEL staining in the spinal cord tissue**. Immunohistochemical analysis for Fas-ligand show positive staining localized in the inflammatory cells in the injured area from PPAR-αWT mice (panel A b, g). The intensity of the positive staining for Fas-ligand was markedly increased in tissue section obtained from injured-PPAR-αKO mice (panel A e, g). No positive staining for Fas-ligand was observed in tissue section from simvastatin-treated PPAR-αWT mice (panel A c, g). The genetic absence of the PPAR-α significantly blocked the effect of simvastatin treatment (panel A f, g). To test whether the tissue damage was associated with cell death by apoptosis, we measured TUNEL-like staining in the injured tissue. No apoptotic cells were detected in the spinal cord from sham-PPAR-αWT mice (panel B a, d and g). Tissue sections from PPAR-αWT animals at 24 h after SCI demonstrate a marked appearance of dark brown apoptotic cells (panel B b, g). Tissue sections from PPAR-αKO animals at 24 h after SCI demonstrate a marked appearance of dark brown apoptotic cells (panel B b, g). The genetic absence of the PPAR-α receptor significantly blocked the effect of simvastatin (panel B f, g). Densitometry analysis of immunocytochemistry photographs (n = 5 photos from each sample collected from all mice in each experimental group) for Fas-ligand from spinal cord tissues was assessed. Data are expressed as % of total tissue area. This figure is representative of at least 3 experiments performed on different experimental days. *P < 0.01 *vs*. Sham; °*P *< 0.01 *vs*. SCI-WT group; °°*P *< 0.01 *vs*. simvastatin-treated WT group. ND: not detectable.

Sections of spinal cord from sham PPAR-α mice did not stain for Fas-ligand (Figure 6Aa, Ad and see densitometry analysis Ag). Spinal cord sections obtained from vehicle-treated SCI-WT mice exhibited positive staining for Fas-ligand (Figure 6Ab, see densitometry analysis Ag) mainly localized in inflammatory cells as well as in nuclei of Schwann cells in the white and gray matter of the spinal cord tissues. In SCI-injured PPAR-αKO mice, the staining for Fas-ligand was visibly and significantly increased (Figure 6Ae, see densitometry analysis Ag) in comparison with WT mice. Section from simvastatin-treated WT mice did not reveal any positive staining for Fas-ligand (Figure 6Ac, see densitometry analysis Ag). The genetic absence of the PPAR-α receptor significantly blocked the effect of the simvastatin on Fas-ligand expression (Figure 6Af, see densitometry analysis Ag).

### Role of PPAR-α in simvastatin-induced inhibition on apoptosis

To test whether the tissue damage was associated with cell death by apoptosis, we measured TUNEL-like staining in the injured tissue. Almost no apoptotic cells were detected in the spinal cord sections from sham PPAR-α mice (Figure 6Ba, Bd see number of TUNEL positive cells Bg). In addition, tissue sections obtained from WT animals at 24h after SCI demonstrate a marked appearance of dark brown apoptotic cells and intercellular apoptotic fragments (Figure 6Bb, see number of TUNEL positive cells Bg). In SCI-injured PPAR-αKO mice, the presence of apoptotic cell (Figure 6Be, see number of TUNEL positive cells Bg) was visibly and significantly increased in comparison with the PPAR-αWT mice. No apoptotic cells were observed in the section from simvastatin-treated PPAR-αWT mice (Figure 6Bc, see number of TUNEL positive cells Bg). The genetic absence of the PPAR-α receptor significantly blocked the effect of simvastatin (Figure 6Bf, see number of TUNEL positive cells Bg).

### Immunohistochemistry for Bax and Bcl-2

**Figure 7:**
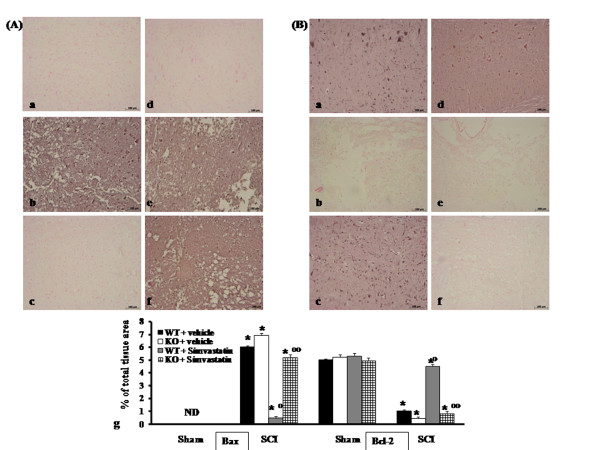
**Immunohistochemistry for Bax and Bcl-2**. Spinal cord tissue were taken in order to determine the immunohistological staining for Bax and Bcl-2. Sections from sham PPAR-αWT mice did not stain for Bax (panel A a, d and g), whereas spinal cord tissue sections obtained from SCI-PPAR-αWT mice exhibited a positive staining for Bax (panel A b, g). The intensity of the positive staining for Bax was markedly increased in tissue section from injured-PPAR-αKO mice (panel A e, g). Spinal cord section from simvastatin-treated PPAR-αWT mice did not reveal any positive staining for Bax (panel A c, g). The genetic absence of the PPAR-α receptor significantly blocked the effect of the simvastatin on Bax expression (panel A f, g). In addition, spinal cord sections from sham-PPAR-αWT mice demonstrated Bcl-2 positive staining (panel B a, d and g), while in spinal cord tissue sections obtained from SCI-PPAR-αWT (panel B b, g) and from SCI-PPAR-α mice (panel B e, g), the staining for Bcl-2 were significantly reduced. The loss of positive staining for Bcl-2 was significant attenuated in the spinal cord from simvastatin-treated PPAR-αWT mice (panel B c, g). The genetic absence of the PPAR-α significantly blocked the effect of the simvastatin on the loss of Bcl-2 staining (panel B f, g). Data are expressed as % of total tissue area. This figure is representative of at least 3 experiments performed on different experimental days. *P < 0.01 *vs*. Sham; °*P *< 0.01 *vs*. SCI-WT group; °°*P *< 0.01 *vs*. simvastatin-treated WT group. ND: not detectable.

Samples of spinal cord tissue were taken at 24 h after SCI in order to determine the immunohistological staining for apoptotic protein family. Sections of spinal cord from sham PPAR-α mice did not stain for Bax (Figure 7Aa, Ad and see densitometry analysis g) whereas spinal cord tissue sections obtained from SCI- WT mice exhibited a positive staining for Bax (Figure 7 Ab, see densitometry analysis g). In SCI-injured PPAR-αKO mice, the staining for Bax was visibly and significantly increased (Figure 7Ae, see densitometry analysis g) in comparison with the PPAR-αWT mice. Section from simvastatin-treated PPAR-αWT mice did not reveal any positive staining for Bax (Figure 7Ac, see densitometry analysis g). The genetic absence of the PPAR-α receptor significantly blocked the effect of the simvastatin on Bax expression (Figure 7Af, see densitometry analysis g).

In addition, spinal cord sections from sham WT PPAR-α mice demonstrated Bcl-2 positive staining (Figure 7Ba, Bd and see densitometry analysis g) while in spinal cord tissue sections obtained from SCI-WT mice the staining for Bcl-2 were significantly reduced (Figure 7Bb, see densitometry analysis g). In SCI-PPAR-αKO mice, the loss of staining for Bcl-2 was visibly and significantly increased (Figure 7Be, see densitometry analysis g) in comparison with the WT mice. The loss of positive staining for Bcl-2 was significant attenuated in the spinal cord tissues from simvastatin-treated PPAR-αWT mice (Figure 7Bc, see densitometry analysis g). The genetic absence of the PPAR-α receptor significantly blocked the effect of the simvastatin on the loss of Bcl-2 staining (Figure 7Bf, see densitometry analysis g).

### Role of PPAR-α in simvastatin-mediated inhibition of SCI-induced NF-κB activation

Most inflammatory mediators, including iNOS, COX-2, IL-1β and TNF-α are controlled by NF-κB, a transcription factor important in inflammatory process, which is kept inactive by IκB [38-40]. Moreover, activation of NF-κB transactivation potential is increased by phosphorylation of the p65 subunit [41]. We performed experiments to evaluate the possible effect of simvastatin on SCI-induced NF-κB potential activation in WT and PPAR-αKO mice at twenty-four hours after trauma.

In sham groups there were no significant differences after simvastatin treatment between PPAR-αWT and PPAR-αKO mice. On the other hand in SCI groups there was a significant reduction of cytoplasmatic IκB-α expression compared to sham groups.

**Figure 8:**
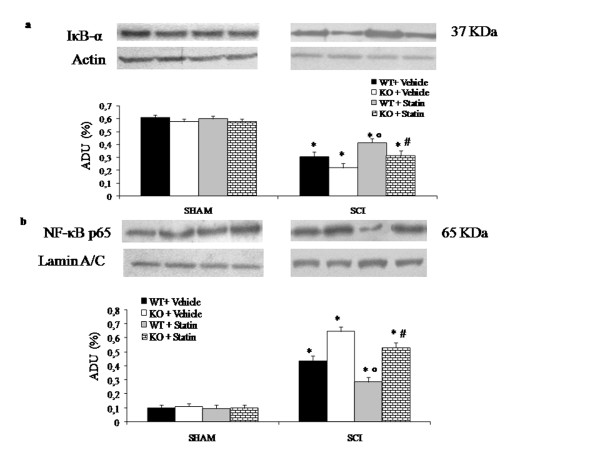
**Effect of PPAR-α on the anti-inflammatory property of simvastatin on NF-κB activation after SCI**. Citoplasmatic IκB-α and nuclear p65 NF-κB levels were detected in the spinal cord from sham and injured-PPAR-αKO mice and PPAR-αWT mice treated or not with simvastatin. A basal level of IκB-α was detected in the spinal cord tissues from sham WT mice (a) and from sham PPAR-α KO mice (a), whereas IκB-α levels were substantially reduced after SCI and significantly in PPAR-αKO mice (a). Simvastatin treatment, instead, increased IκB-α levels in both mice group but significantly more in WT treated mice (a). In addition, SCI caused a significant increase in nuclear NF-κB p65 compared to the sham-operated mice (b) and these increase was higher in SCI PPAR-αKO mice (b) than sham PPAR-αWT mice (b). Simvastatin treatment (10 mg/kg at 1 and 6 h after SCI induction) significantly reduced NF-κB p65 levels much more in WT treated mice (b) than PPAR-αKO mice (b). Polyclonal actin and lamin were used as internal control. The bars of each western blot are represented a densitometry analysis expressed as mean ± S.E.M. of three different experiments. *P < 0.01 *vs*. sham; °*P *< 0.01 *vs*. SCI-WT group; #*P *< 0.01 *vs*. simvastatin-treated group.

In untreated SCI mice, IκB-α levels were significantly reduced in PPAR-αKO mice respect to PPAR-αWT mice (Figure 8a). Simvastatin administration prevented the SCI-induced IκB-α degradation in PPAR-αWT mice while in PPAR-αKO the statin could not prevent IκB-α degradation (Figure 8a).

Moreover, NF-κB p65 levels in the nuclear fractions from spinal cord tissue were also significantly increased in SCI groups compared to sham groups (Figure 8b). After SCI untreated PPAR-α KO mice showed NF-κB levels higher than PPAR-αWT mice (Figure 8b). Simvastatin treatment (10 mg/kg) significantly reduced the SCI-induced NF-κB activation in PPAR-αWT mice more than in PPAR-αKO (Figure 8b). Therefore the absence of PPAR-α gene interferes with the protective effect of statin (Figure 8a, b).

## Discussion

The CNS is sensitive to mechanical injuries causing permanent functional deficits such as the case of patients who have SCI. The mechanical forces imparted to the spinal cord cause immediate tissue disruption, with a direct axonal and neuronal injury, inducing the death of a number of neurons that cannot be recovered and regenerated. Moreover, neurons continue to die for hours after SCI due to several mechanisms including excitotoxicity, vascular abnormalities and inflammatory response that can contribute to evolution of spinal cord secondary injury [42].

Post-traumatic inflammation is determined by a number of cellular and molecular events [43]. Leukocytes are directly involved in the pathogenesis and extension of SCI [43,44].

In the present paper we show that the absence of PPAR-α gene, in PPAR-α KO mice, results in a reduced anti-inflammatory response to simvastatin treatment in an experimental model of SCI. These results are in agreement with a previous observations indicating that the acute anti-inflammatory effect of simvastatin occurs via PPARα by a mechanism involving inhibition of PKCα inactivation of PPARα transrepression activity [28].

Clinical trials and in vitro studies have shown that statins and PPAR-α agonists share anti-inflammatory properties by regulating inflammatory-response genes [45,46]. In particular, the in vivo anti-inflammatory effects of simvastatin on footpad swelling and neutrophil recruitment in air pouch-bearing mice already occur within 1 hour after a single oral administration, indicating that the PPARα-dependent anti-inflammatory effects of simvastatin occur rapidly [28].

PPAR-α is also able to directly mediate anti-inflammatory effects and it has been shown that its agonist-induced activation inhibits a number of inflammatory mechanisms including TNF-α production, iNOS and COX-2, adhesion molecules expression as well cell infiltration in the tissues [35,47].

Based on these observations we performed studies in the attempt to determine whether the presence and/or the stimulation of PPAR-α could enhance the simvastatin anti-inflammatory efficacy. For that purpose we used an experimental model of SCI induced by extradural compression of the spinal cord (T6-T7) using an aneurysm clip with a closing force of 24 g via a four-level T5-T8 laminectomy, performed in WT and PPAR-αKO mice.

There is a large body of evidence showing that the production of reactive oxygen and nitrogen species play key roles in the development of secondary neuronal damage of SCI [48-52].

Not only peroxynitrite (ONOO^-^) was detected in spinal cord tissues from rats following traumatic injury [52,53], but ONOO^- ^donor administration directly into the rat spinal cord has been shown to cause neuronal cell death and neurological deficits [48,49]. Previous reports have demonstrated that ONOO^- ^is toxic for neurons *in vitro *[54] and recently it have been also established that primary spinal cord neurons also undergo cell death following ONOO^- ^treatment [51]. ONOO^- ^is known to mediate several potentially destructive chemical reactions, including tyrosine nitration and lipid peroxidation [55,56]. Mitochondrial respiration is directly inhibited by ONOO^- ^and is an early marker of its cytotoxic effects [54,57]. Several antioxidants showed neuroprotection in SCI and in associated conditions like oxidative stress and inflammation [35,48,51].

In the present study we clearly demonstrate that when WT and PPAR-αKO mice were treated with simvastatin a significant inhibition of nitrotyrosine formation was observed in WT but not in PPAR-αKO mice.

In addition, one consequence of increased oxidative stress is the activation and inactivation of redox-sensitive proteins [58]. Recent evidence suggests that the activation of NF-κB may also be under the control of oxidant/antioxidant balance [59].

In the present study we demonstrated that PPARα and simvastatin were synergic in negatively interfere with the NF-κB signalling transduction. In fact in spinal cord tissues simvastatin treatment was able to significantly reduce the NF-κB activation. The important anti-inflammatory role of PPARα was evident in PPARα lacking mice where the NF-κB activity was less reduced compared to PPARαWT mice. These data confirmed our previous studies that explained the anti-inflammatory effect of PPARα through an inhibition of NF-κB pathway [60,61]. There is evidence that production of pro-inflammatory cytokines, such as TNF-α and IL-1β, is important to induce local and systemic inflammation and that production of this cytokine can be inhibited by treatment with statin [28,62].

Benani and colleagues reported in a recent work [63] that PPAR-α expression was less expressed in the gray matter, while a high expression was observed in some cells in the white matter, especially in astrocytes [63]. The presence of PPAR-α in astrocytes suggested at these authors that this isoform modulates central inflammation, possibly by regulation of cytokine production by astrocytes. This function of anti-inflammatory factor has been demonstrated outside the CNS, as an inflammation induced by leukotriene B4, a natural inflammation mediator, was prolonged in PPAR-α null mice as compared to control animals [64,65]. In the present study we demonstrate that when WT and PPAR-αKO mice were treated with simvastatin a significant inhibition of TNF-α and IL-1β level was measured in WT but not in PPAR-αKO mice.

NF-κB activation is crucially involved in Fas-Ligand expression induced by DNA-damaging agents, such as genotoxic drugs and ultraviolet (UV) radiation [66]. FasL plays a central role in apoptosis induced by a variety of chemical and physical insults [67]. Recently, it has been pointed out that FasL signalling plays a central role in spinal cord injury [68] and cell death induced by reactive oxygen species (ROS) depends on FasL expression mediated by redox sensitive activation of NF-κB [69]. We confirm here that SCI leads to a substantial activation of FasL in the spinal cord tissues which likely contributes in different capacities to the evolution of tissues injury. In the present study, we found that when WT and PPAR-αKO mice were treated with simvastatin a significant inhibition of FasL activation was evaluated in WT but not in PPAR-αKO mice.

Recent studies have also demonstrated ROS induce apoptosis in an early and likely causal event that contributes to the spinal cord motor neuron death [70] following SCI [71]. Apoptosis is an important mediator of secondary damage after SCI, it incurs its affects through at least two phases: an initial phase, in which apoptosis accompanies necrosis in the degeneration of multiple cell types and a later phase, which is predominantly confined to white matter and involves oligodendrocytes and microglia [72]. Chronologically, apoptosis initially occurs 6 hours post-injury at the lesion centre and last for several days associated with the steadily increased number of apoptotic cells in this region. An important intracellular signal transduction pathway that leads to apoptosis after SCI involves activation of the caspases, in particular, caspase-3 [73].

Has been demonstrated that PPAR-α suppress the apoptosis in hepatocytes [74], we have also recently demonstrated that exogenous PPAR-α ligand inhibits apoptotic cell death in spinal cord tissues from PPAR-αWT mice subjected to SCI. In the present study, with immunohistological staining, we clearly demonstrate that WT mice treatment with simvastatin induced a significant inhibition of apoptotic process by reducing the expression of pro-apoptotic gene like Bax and preventing the loss of anti-apopoptotic gene Bcl-2. Notably, the absence of functional PPAR-α receptor in PPAR-αKO mice inhibits the effect of simvastatin treatment on the apoptotic process.

Moreover, previous studies showed that PPAR-α agonists exert some anti-inflammatory activity [35,47].

Statins are competitive inhibitors of HMG-CoA reductase, the rate-limiting step in cholesterol synthesis thus decreasing endogenous cholesterol synthesis. In addition to their potent action on plasma lipid concentrations, statins exert pleiotropic activities such as that antiplatelet, antioxidant, anti-inflammatory and immunomodulatory [75,76]. Just for these pleiotropic effects statins are considered a valid therapeutic approach for the different and numerous pathways involved in the pathogenesis of sepsis. In fact, clinical data obtained from retrospective database and observational studies suggested that statins may be helpful in sepsis [77].

Moreover a large and growing body of evidences also suggest beneficial therapeutic activities of statins in immune and inflammatory diseases such as multiple sclerosis, Alzheimer's disease, ischemic stroke, transplant rejection, rheumatoid arthritis, and asthma [10,11].

The efficacy of simvastatin treatment in cardiovascular, inflammatory and autoimmune diseases is an important therapeutic subject and while some patients obtain clinical improvements from treatment, others are not responsive or even resistant to therapy. The reasons of response or not response to statin therapy are not fully understood and molecular mechanisms.

## Conclusions

In conclusion, results here discussed confirm a new mechanism contributing to determine the full statin efficacy and suggest future studies aimed to analyze the possible relevance of PPAR-α in other human inflammatory diseases such as SCI.

## List of abbreviations used

IL-1β: interleukin 1β; iNOS: inducible nitric oxide synthase; MPO: myeloperoxidase; i.p.: intraperitoneally; PBS: Phosphate buffered saline; PMN: polymorphonuclear leukocyte; PMSF: phenyl-methyl sulfonyl fluoride; ROS: reactive oxygen species; SDS: sodium dodecyl sulphate.

## Competing interests

The authors disclose any financial competing interests and also any non-financial competing interests that may cause them embarrassment were they to become public after the publication of the manuscript

## Authors' contributions

Research design: EE, BR, SC, AC, PB; In vivo esperimenta: EM, DI, IP; Western blot analysis: EE, MD; Data analysis: BR, AC, SC; Writing of manuscript: EE, BR, SC; All authors read and approved the final manuscript.
